# Antimicrobial Properties of Secondary Metabolites Produced by Halomonas sp.: A Halophilic Bacterium

**DOI:** 10.7759/cureus.69633

**Published:** 2024-09-18

**Authors:** Keerthana Perumal, Jayaprakash Seenuvasan, Manivannan Nandhagopal

**Affiliations:** 1 Department of Microbiology, Saveetha Medical College and Hospital, Saveetha Institute of Medical and Technical Sciences, Saveetha University, Chennai, IND

**Keywords:** antimicrobial activity, anti-oxidant potential, gc-ms analysis, halomonas sp, secondary metabolites

## Abstract

Aim

It is unknown whether the halotolerant bacterium *Halomonas* sp. produces a range of secondary metabolites with antimicrobial qualities. In the past few years, there has been a growing interest in the biotechnological capability of halophilic bacteria for the production of antimicrobial compounds.

Materials and methods

The current review intended to assess the antibacterial and antifungal properties of microbial metabolites, explicitly those produced as secondary metabolites by a putative halophilic bacterium. First, phenotypic and genotypic identification were used to identify and confirm the obtained potent halophilic bacterium as *Halomonas* sp., and its antioxidant properties and biological compatibility were studied.

Results

The extracellular metabolites that were obtained exhibit a moderation zone of inhibition against 11 mm of *Staphylococcus aureus*, 12 mm of *Pseudomonas aeruginosa*, and 11 mm of *Candida albicans*. The optimal inhibitory concentration for *S. aureus* and *P. aeruginosa* is 256 µg/mL, while the minimum inhibitory concentration (MIC) for *C. albicans* is 128 µg/mL. The antioxidant property of crude metabolites indicates that 100% scavenging at 512 µg/mL, and the blow at 256 µg/mL, are not reasonable levels of antioxidant activity.

Conclusion

Secondary metabolites appear to be highly biologically compatible, as there is no hemolytic activity at any of the tested concentrations. According to the study, *Halomonas* sp.'s secondary metabolites could be a source for the synthesis of novel antimicrobial compounds.

## Introduction

Bacteria classified as halophilic grow best in environments with high concentrations of salt. Extreme environments, such as saltwater ponds, saline lakes, hypersaline soils, arid soils, and preserved food, can all support the survival and growth of these bacteria [1]. The development and metabolic activity of halophilic bacteria depend heavily on their capacity to endure a range of environments [2]. The rise in antibiotic resistance has made it harder to find antibiotics from different sources in recent years. Therefore, according to Deepalaxmi and Gayathri (2018) [3], halophilic bacteria may offer an alternative source of antimicrobial compounds. According to Gasperotti et al. (2018) [4], halophilic bacteria such as *Halomonas* sp. are known to flourish in exceptionally salinized conditions and have the capacity to produce stable secondary metabolites with antimicrobial qualities like 1-acetyl-β-carboline, diketopiperazines (DKPs) cyclo(2-OHPro-Phe), macrolactin and succinoyl macrolactones. These secondary metabolites have demonstrated a significant role in the fields of biotechnology and pharmaceuticals. They include compatible solutes, enzymes, carotenoid pigments, and biopolymers [5,6]. There hasn't been much research done on the antimicrobial qualities of the extracellular and intracellular metabolites that *Halomonas denitrificans* produces. On the other hand, recent research has demonstrated that antimicrobial metabolites are produced by halotolerant *Bacillus licheniformis* strains that were isolated from saline and hypersaline environments [7]. Both halophilic and non-halophilic bacteria are members of the *Gammaproteobacteria* class, which includes the *Halomonadaceae*. This includes halophilic and halotolerant species like *Modicisalibacter*, *Halomonas*, *Cobetia*, *Chromohalobacter*, and *Kushneria*. Conversely, non-halophilic bacteria are present in closely related halophilic members of the family, such as *Zymobacter*, *Carnimonas*, and *Halotalea* [8].

The fields of microbial biotechnology and related research have opened up new avenues for the industrial use of ambient microbes that generate bioactive compounds and secondary metabolites. Microbes are widely distributed and beneficial to sustainable development in many fields, such as pharmaceuticals, industry, agriculture, and other related areas where pathogen control is needed. The rod-shaped, gram-negative, non-spore-forming *Halomonas* organisms are mostly dependent on respiratory metabolism. They take electrons from either oxygen or nitrate; some species are denitrifiers, and some have fermentative metabolism. While some species of *Halomonas* may be considered halotolerant, most species show a preference for mild salinities. Microbial secondary metabolites are substances that microorganisms produce. Rather than being necessary for their basic metabolic processes, such as growth and reproduction, these metabolites are vital to their survival and ability to adapt to various environmental conditions [9]. The microbial cells of *Halomonas* sp. produce organic compounds known as intracellular metabolites, which are indicative of biological activity in the organism. These metabolites can be produced by a variety of metabolic pathways and have roles in the cell [10]. They are crucial for a number of cellular functions, including the synthesis of macromolecules, the production of energy, and the preservation of cellular homeostasis [11]. Additionally, the intracellular metabolites from halophilic bacteria also exhibit antimicrobial properties [12]. *Bacillus halophilus* sp. BS3 and* Kocuria marina* BS-15 produce polymeric biosurfactants with anticancer properties, while other halophilic bacteria produce lipopeptide-like biosurfactants [13,14]. Therefore, the promising field for drug discovery and development may be the antimicrobial qualities of the extracellular, intracellular, and lipopeptides of halophilic bacteria produced by *H. denitrificans*. Additional investigation into these characteristics may result in the creation of novel antibiotics and all-natural preservatives, in addition to other biotechnological uses. In general, microbial metabolites are significant for clinical and therapeutic uses. Therefore, investigating these microbial metabolites from environmental microorganisms, such as halophilic bacteria, may have a wide range of applications.

## Materials and methods

Halophilic isolate

The Department of Microbiology, Saveetha Medical College and Hospital, Chennai, India, provided the strong halophilic bacterial isolate, which was obtained from the Bio-control and Microbial Product Lab. It was kept in a nutrient agar slant medium with 8% NaCl and maintained at 7°C for additional analysis.

Clinical pathogens

The antimicrobial activity assay involved the use of five distinct pathogenic bacteria: *Candida albicans*, *Pseudomonas aeruginosa*, methicillin-resistant *Staphylococcus aureus *(MRSA), *Escherichia coli*, and *Enterococcus faecalis*. For 30 days, the pathogenic organisms were regularly cultivated and sub-cultured in Mueller-Hinton Agar (MHA) medium (HIMEDIA M173-500G; HiMedia, Thane, Maharashtra, India) to maintain cell viability, while *C. albicans* was kept in Sabourad Dextrose Agar (SDA) medium (HiMedia).

Bacterial DNA isolation and amplification of 16s rRNA

The DNA isolation and 16S rRNA sequencing of potent halophilic bacteria was performed at Eurofins Genomics (Bangalore, India). The halophilic bacteria were cultured in Nutrient Broth (NB) medium. Following a 24-hour growth period, the bacterial cells were cleaned with phosphate-buffered saline (PBS). Lysis buffer was then added to the bacterial cells to facilitate the chemical lysis process that released the DNA. After adding the binding buffer to the lysate to denature the proteins and safeguard the DNA, the DNA was bound to silica. It was added to a spin column with a silica membrane; in the presence of a high concentration of salt, DNA will bind to the membrane. To eliminate impurities, the silica membrane was cleaned using several washing buffers. Water was utilized as the elution buffer to remove the DNA from the silica membrane, and agarose gel electrophoresis was used to examine the resultant DNA sample. Until its next use, the extracted DNA was stored at -20°C or -80°C.

Amplification of DNA

Using universal primers, the extracted DNA from the bacterial sample was amplified by polymerase chain reaction (PCR) to produce a copy of the 16S rRNA gene region. The conserved region was intensified using primers 27F (5'-AGAGTTTGATCMTGGCTCAG-3') and 1492R (5'-GGTTACCTTGTTACGACTT-3'). The PCR mixture is first denatured at 95°C for five minutes. After that, there are 35 cycles of denaturation (at 95°C for 30 seconds), annealing (at 55-60°C for 30 seconds), and extension (at 72°C for 60 seconds), culminating in a final extension of 10 minutes at 72°C.

Amplification and genomic analysis of 16S rRNA

Sequencing

The refined PCR result was sent to the European Sequencing Center in Bangalore, a sequencing service provider, for Sanger sequencing. The File Alignment Sequence Transfer (FAST) format sequencing data was obtained through the use of the European Molecular Biology Open Software Suite (EMBOSS) Merger, which merged the forward and reverse primers (bioinformatics.nl). The obtained nucleotides were run through the National Center for Biotechnology Information's (NCBI's) Basic Local Alignment Search Tool (BLAST) analysis. Nucleotide BLAST analysis was used for data analysis, and the data was downloaded as a Fast-All (FASTA) file. Neighbor-Joining analysis of the evolutionary history was performed [15]. The evolutionary history of the taxa was examined using the bootstrap consensus (1000 replicates) method, as described by Felsenstein (1985) [16]. Using the Likelihood method, as outlined by Tamura et al. (2004) [17], branches corresponding to evolutionary distances were calculated, and evolutionary analyses were carried out in Molecular Evolutionary Genetics Analysis 11 (MEGA11) [18].

Synthesis and recovery of secondary metabolites

The production of microbial metabolites was carried out in an NB medium with 8% NaCl. The obtained bacterial culture was cultivated for nine days in 250 milliliters of NB medium, or until a change in the growth medium's color was noticed. Centrifugation was used to gather the cell-free supernatant after growth and filtration followed. After adding 1/2 of the ethyl acetate to the obtained culture filtrate and thoroughly mixing it in a separating funnel, the ethyl acetate was separated, collected, and used to conduct the crude metabolites before being stored for additional research.

Antimicrobial test: well diffusion technique

The secondary metabolites of *Halomonas* sp. were assessed using the well diffusion assay against the following five pathogens: *C. albicans*, *P. aeruginosa*, *E. coli*, MRSA, and *E. faecalis*. The pathogens were grown as a mother inoculum using sterile Mueller-Hinton Broth (MHB), and their optical density (OD) was adjusted to 0.4 at 600 nm. On sterile MHA medium plates, the pathogens were subsequently dispersed using lawn culture techniques. After making wells with a cork borer, 100 µL of crude metabolites were added to the MHA plates. Using a zone scale, the zones of inhibition were identified after a 16-hour incubation period at 37°C. The experiments were conducted in three sets.

Minimum inhibitory concentration (MIC)

Regulations from the European Committee on Antimicrobial Susceptibility Testing (EUCAST) and the Clinical and Laboratory Standards Institute (CLSI) were followed in determining the MIC. The first 10 wells of microtiter test plates were filled with crude metabolites, Ci, at concentrations of Ci = (512, 256, 128, 64, 32, 16, 8, 4, 2, 1) mg/L. Furthermore, Pi - which stands for 5 μL of human pathogens - was added to every well, with the exception of the negative control. The plates were incubated at 37°C for 16 hours. Following incubation, the volume of newly made MTT (VMTT) was added, with VMTT equal to 10 μL and the concentration of MTT (CMTT) equal to 5 mg/mL. After covering the wells with aluminum foil, they were left alone for the time for MTT incubation (tMTT) = 1 hour. After that, a volume of dimethyl sulfoxide (VDMSO) (solubilization solution) was added; VDMSO = 100 μL; the mixture was then left for 15 to 30 minutes to fully solubilize. In an enzyme-linked immunosorbent assay (ELISA) reader, the OD at a wavelength of λ = 595 nm was used to calculate the percentage of cell death (PD) using the following formula.

$\text{Cell Viability (\%)} = \left( \frac{\text{Absorbance of treated cells}}{\text{Absorbance of control cells}} \right) \times 100$

The results observed were recorded and contrasted with the effectiveness of commercially available antibiotics, as described by Rajagopal et al. (2023) [19].

Assay for antioxidant activity using 2,2-diphenyl-1-picrylhydrazyl (DPPH)

The DPPH assay method, described by Malterud (1993) [20], was used to measure the DPPH scavenging activity (SA) of crude metabolites from *Halomonas* sp. In the beginning, the concentration range of the crude metabolites, Ci, was diluted to include Ci = (512, 256, 128, 64, 32, 16, 8, 4, 2, 1) mg/L. After adding 100 μL of DPPH, the mixture was left to incubate for 20 minutes at room temperature in the dark. Next, a negative control was added, with Vmethanol = 100 μL, containing 99% methyl alcohol. Serving as the positive control was ascorbic acid. At λ = 517 nm, the absorbance (A) was measured. The percentage (%) of DPPH radical SA was determined using the formula:

$\text{Antioxidant Activity} (\%) = \frac{\text{Absorbance of Control} - \text{Absorbance of Sample}}{\text{Absorbance of Control}} \times 100$

The results were evaluated and contrasted using the methodology of Ralte et al. (2022) [21].

Hemolytic activity

Human blood cells were used to test the biological compatibility of secondary metabolites from *Halomonas* sp., specifically the recently extracted human blood cells from the participants, followed by three PBS washes. PBS was used as the negative control, and 1% Triton X-100 was used as the positive control. The various concentrations of secondary metabolites (512, 256, 128, 64, 32, 16, 8, 4, 2, and 1 μg/mL) were diluted in 800 µL of PBS solution, and 200 μL of blood sample was added to each microcentrifuge tube separately. The tubes were centrifuged at 5000 rpm for seven minutes and allowed to incubate for an hour at 37°C. The amount of hemoglobin released was calculated using absorbance measurements made at 570 nm [22].

$\text{Hemolysis} (\%) = \frac{\text{Absorbance of Sample} - \text{Absorbance of Control}}{\text{Absorbance of Positive Control} - \text{Absorbance of Control}} \times 100$

These investigations were done twice and results are expressed by mean ± standard deviation (±SD).

Gas chromatography-mass spectrometry (GC-MS) examination of halophilic bacteria's secondary metabolites

A study on the molecules found in *Halomonas* sp. secondary metabolites was carried out using the GC Ultra and DSQ II model mass spectrometers. The engine vacuum strain (P), connection point temperature (T_connection), source temperature (T_source), and injector port temperature (T_injector) were all set on the instrument to 250°C, 200°C, and 250 psi, respectively. The oven temperature (T_oven) was programmable, with changes specified as follows. The DB-35 MS non-polar section measured 0.25 μm in inner diameter and 0.25 mm in outer diameter. Helium was used as the carrier gas at a rate of 1 mL/min. The mass spectrometer was set up to find fragments between 50 Da and 650 Da in mass. The ionization energy (IE) of the MS was set at -70 eV, and it included a pre-filter to remove neutral particles. The results were contrasted with the library's reference data.

Statistical analysis

Every experiment was conducted thrice, and the mean ± SDs were used to express the results using Prism software (GraphPad Software Inc., San Diego, CA, USA).

## Results

Morphological and phenotypic characterization of halophilic bacteria

**Figure 1:**
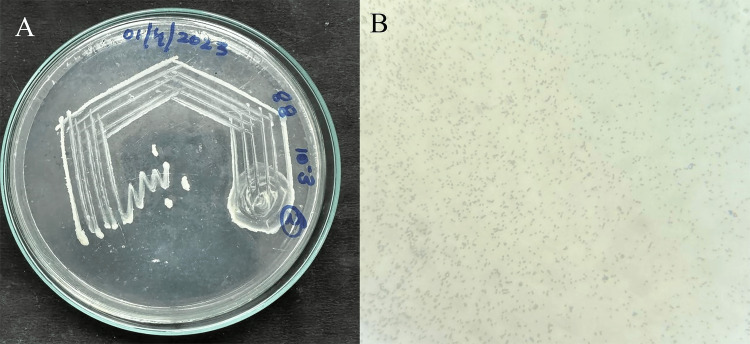
Colony morphology of halophilic bacteria A) Colony morphology of potent halophilic bacterium; B) Gram's staning of halophilic bacterium

In nutrient agar medium with 8% NaCl, the halophilic bacteria's morphology revealed pale yellow, round-shaped, flat, and translucent colonies. A gram-negative rod arrangement was observed under light microscopy following gram staining (Figure 1).

The biochemical tests show that urease is positive, hydrogen sulfide production is negative, oxidase and catalase are positive, and indole is negative.

Molecular identification of bacterial isolates

**Figure 2:**
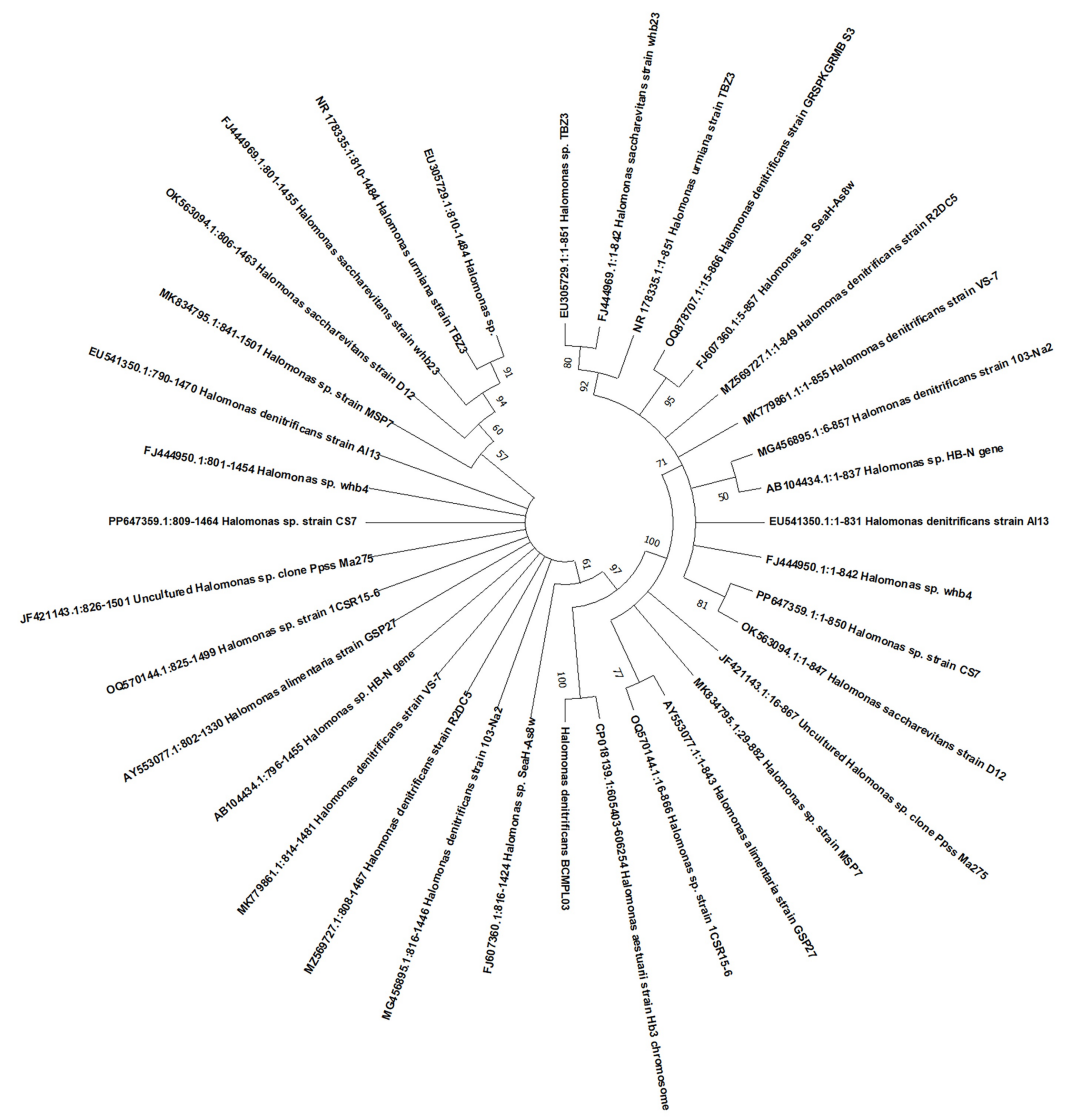
Phylogenetic tree construction of halophilic bacterium

The GenBank NCBI database assigned the DNA sequences of the halophilic bacterial isolates through the use of the BLAST 2.0 program for identification. The halophilic organism most closely resembled *H. denitrificans*, according to the results of the BLAST run. It was followed by *Halomonas shengliensis*, *Halomonas huangheensis*, *Halomonas binhaiensis*, *Halomonas cupid*, *Halomonas stenophila*, and *Halomonas pacifica*. It was confirmed that the potent bacterium belonged to the *Halomonas* sp. (Figure 2).

Antimicrobial activity of microbial metabolites

**Figure 3:**
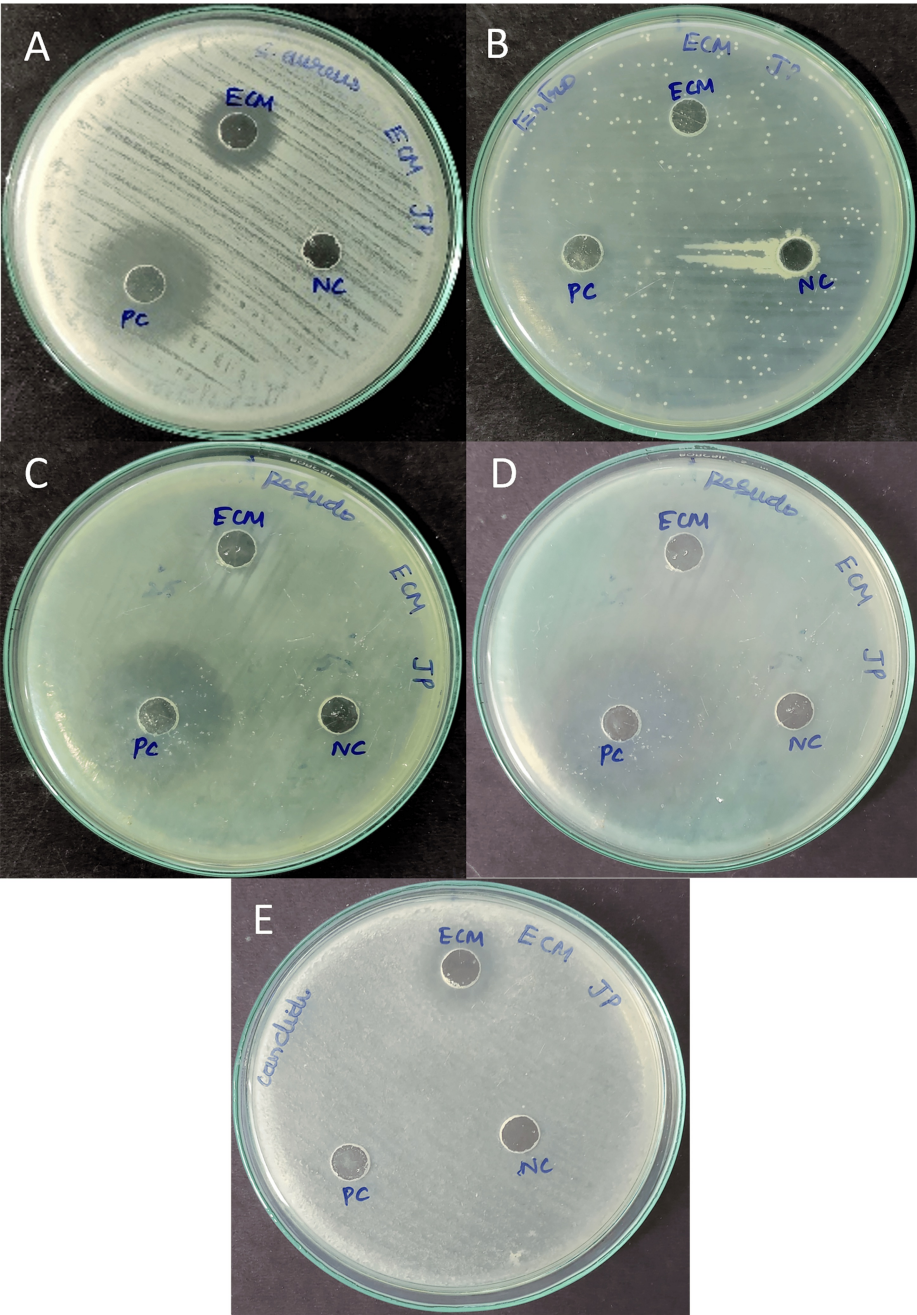
Antimicrobial property of secondary metabolites from Halomonas sp. A) *Staphylococcus aureus*; B) *Enterococcus faecalis*; C) *Escherichia coli*; D) *Pseudomonas aeruginosa*; E) *Candida albicans*

A 14 mm zone of inhibition was observed by the extracellular metabolites of *Halomonas* sp. against both *S. aureus* and *C. albicans*. However, it was unable to demonstrate any antimicrobial activity against *E. coli* and *E. faecalis*. In contrast, *S. aureus*, *P. aeruginosa*, and *E. coli* were all effectively inhibited by the positive control, which consisted of commercially available antibiotics (Figure 3; Table 1).

**Table 1:** Antimicrobial activity of extracellular metabolites produced by Halomonas sp. ECM: Extracellular metabolites; PC: Positive control

Zone of inhibition (mg/mL)									
Gram-positive bacterium				Gram-negative bacterium				Fungal pathogen	
Staphylococcus aureus		Enterococcus faecalis		Escherichia coli		Pseudomonas aeruginosa		*Candida* *albicans*	
ECM	PC	ECM	PC	ECM	PC	ECM	PC	ECM	PC
14	25	-	-	-	27	12	27	13	-

MIC

It was established what *Halomonas* sp.'s MIC was for each of the five clinical pathogens. It was found that the extracellular metabolites from *Halomonas* sp. had a maximum concentration of growth inhibition of 128 mg/L and 256 mg/L, respectively, against *S. aureus*, *C. albicans*, and *P. aeruginosa*. Table 2 shows that no growth inhibition was observed against *E. coli* or *E. faecalis*.

**Table 2:** Minimum inhibitory concentration of extracellular metabolites produced by Halomonas sp. ECM: Extracellular metabolites; PC: Positive control; ND: Not determined

Minimum inhibitory concentration (MIC) (mg/L)									
Gram-positive bacterium				Gram-negative bacterium				Fungal pathogen	
Staphylococcus aureus		Enterococcus faecalis		Escherichia coli		Pseudomonas aeruginosa		Candida albicans	
ECM	PC	ECM	PC	ECM	PC	ECM	PC	ECM	PC
128	2	ND	ND	ND	1	256	2	128	ND

Antioxidant activity of microbial metabolites

**Figure 4:**
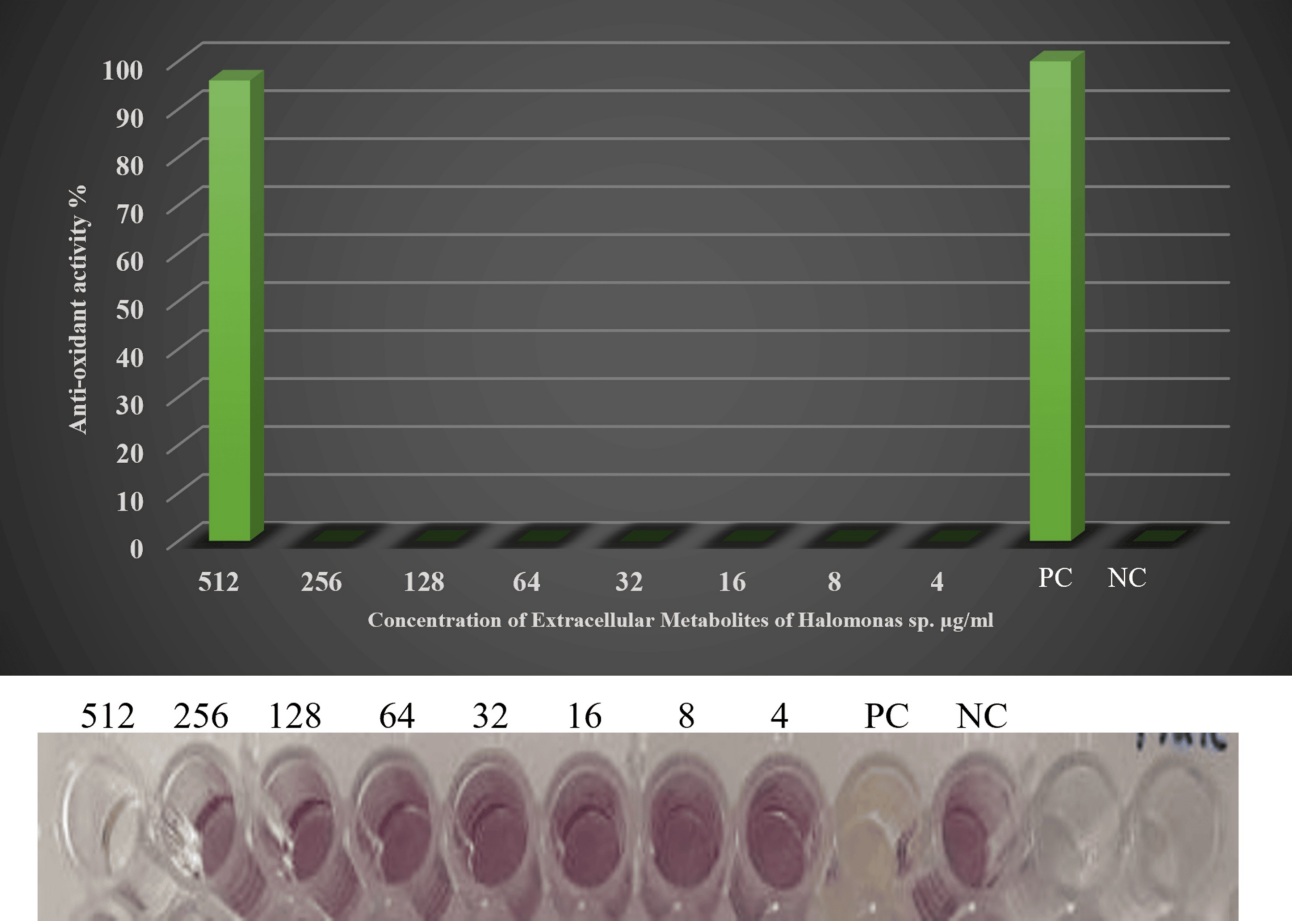
Anti-oxidant activity of secondary metabolites form Halomonas sp. PC: Positive control; NC: Negative control

The extracellular metabolites of *Halomonas* sp. were found to have antioxidant activity that was able to reduce free radicals at a concentration of 512 mg/mL but did not reduce DPPH SA at a concentration of 256 mg/mL (Figure 4).

Hemolytic activity of the microbial metabolites

**Figure 5:**
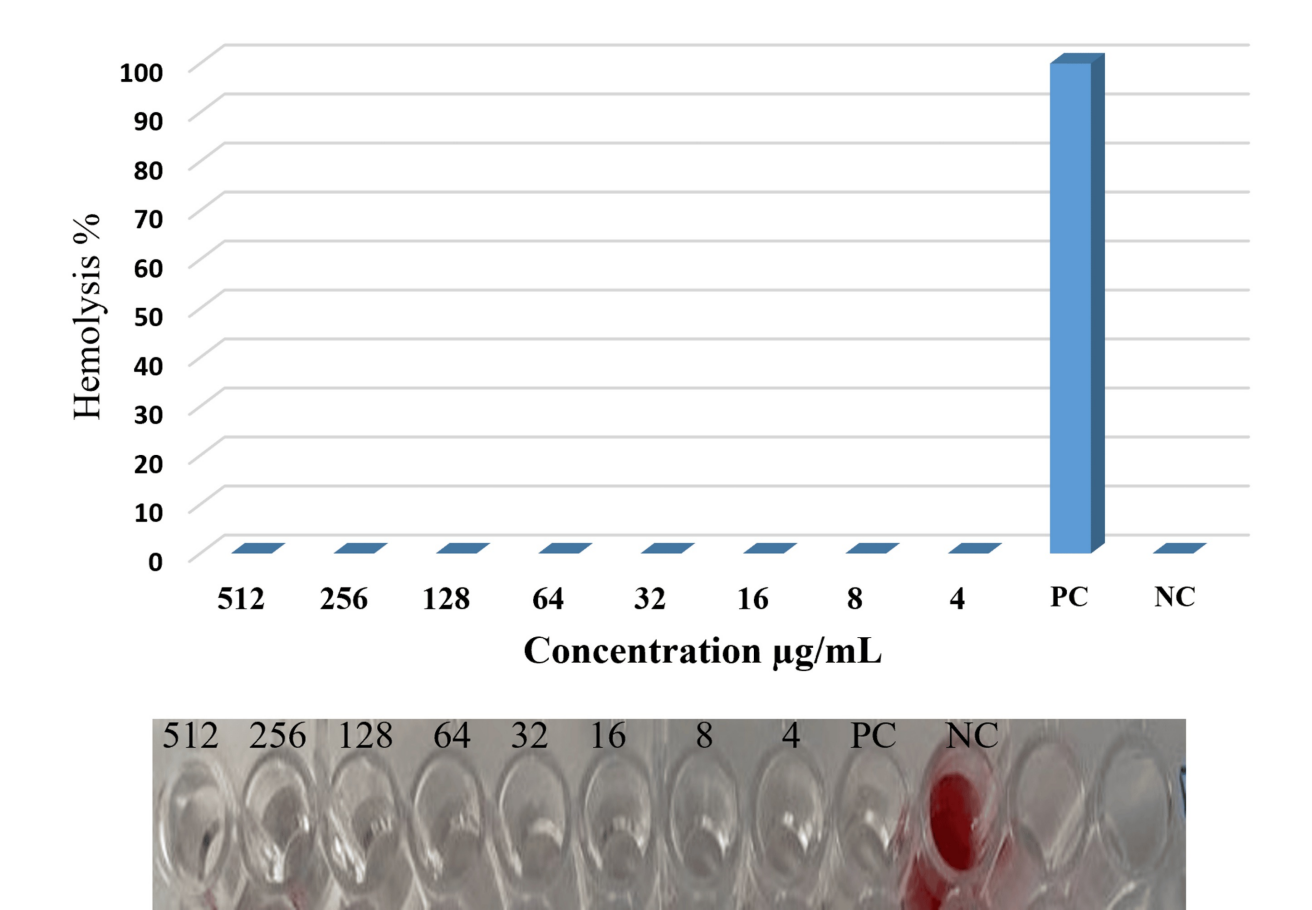
Hemolytic activity of secondary metabolites from Halomonas denitrificans PC: Positive control; NC: Negative control

The effects on red blood cells of varying concentrations of secondary metabolites were evaluated. This information provides a quantitative assessment of hemolytic activity and includes measurements of hemoglobin release or alterations in cell integrity. According to a comparison of the results, there is no hemolytic activity at any of the tested concentrations of the secondary metabolites (Figure 5).

GC-MS analysis

**Figure 6:**
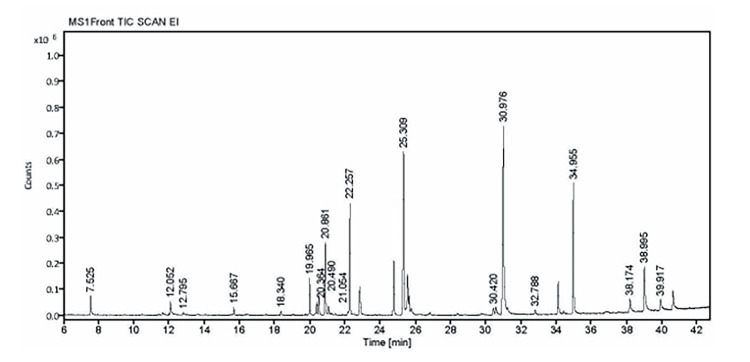
Chromatogram of GC-MS analysis of secondary metabolites GC-MS: Gas chromatography-mass spectrometry

A complex mixture of compounds was discovered by GC-MS analysis of crude metabolites from *Halomonas* sp. (Figure 6).

Propanoic acid, 3-chloro-, 4-formylphenyl ester, with a peak area of 1.31% and a lower probability of 38.75%, is among the substances that have been identified. On the other hand, 2-piperidinone has been detected with a relative peak area of 1.42% and a high identification probability of 84.95%. Another component detected had a probability of 35.27% and a peak area of 0.36%. 2,4-Di-tert-butylphenol was also present, with a probability of 41.1% and a peak area of 0.54%. Many piperazine derivatives, including 3-methyl-6-(1-methylethyl)- and 2,5-piperazinedione, as well as their structural analogs, showed peak areas of less than 1% and probabilities exceeding 70%. Pyrrolo[1,2-a]pyrazine-1,4-dione, hexahydro-, which represented the highest peak area at 20.06% with a 94.37% probability, was one of the noteworthy findings. Other noteworthy compounds included pyrimidine-2(1H)-thione, 4,4,6-trimethyl-1-(1-phenylethyl)-, with a peak area of 2.76% and a 20.61% identification probability, and cyclo, with a peak area of 9.31% and a high probability of 94.78%. Each metabolite found in the Japan Pharmacopeia-Excipients for Microorganisms (JP-ECMs) is thoroughly profiled by the analysis, and Table 3 shows the molecular name, molecular formula, and molecular weight of each metabolite.

**Table 3:** GC-MS analysis of secondary metabolites from Halomonas sp. GC-MS: Gas chromatography-mass spectrometry

Peak	Reaction time	Area %	Name	Probability %	Formula	Mol. wt
1	7.525	1.42	2-piperidinone	84.95	C_5_H_9_NO	99.13
2	12.052	1.31	Propanoic acid, 3-chloro-, 4-formylphenyl ester	38.75	C_10_H_9_ClO_3_	212.02
3	12.795	0.36	Benzeneacetamide	35.27	C_8_HNO	135.07
4	15.667	0.54	2,4-Di-tert-butylphenol	41.1	C_14_H_22_O	206.17
5	18.340	0.42	2,5-piperazinedione, 3-methyl-6-(1-methylethyl)-	87.16	C_8_H_14_N_2_O_2_	170.11
6	19.965	3.17	1,4-diazabicyclo[4.3.0]nonan-2,5-dione, 3-methyl	90.99	C_8_H_12_N_2_O_2_	168.09
7	20.364	1.07	dl-Alanyl-dl-leucine	80.77	C_9_H_18_N_2_O_3_	202.13
8	20.490	1.69	(3S,6S)-3-butyl-6-methylpiperazine-2,5-dione	46.12	C_9_H_16_N_2_O_2_	184.12
9	20.861	5.32	Pyrrolo[1,2-a]pyrazine-1,4-dione, hexahydro-	81.2	C_9_H_10_N_2_O_2_	154.07
10	21.054	0.50	3-Isobutyl-2,5-piperazinedione	73.31	C_8_H_14_N_2_O_2_	170.11
11	22.257	9.31	Cyclo(L-prolyl-L-valine)	94.78	C_10_H_16_N_2_O_2_	196.12
12	25.309	20.06	Pyrrolo[1,2-a]pyrazine-1,4-dione, hexahydro-3-(2-methylpropyl)-	94.37	C_11_H_18_N_2_O_2_	210.04
13	30.420	0.95	2,5-piperazinedione, 3-methyl-6-(phenylmethyl)-	72.01	C_12_H_14_N_2_O2	218.11
14	30.976	28.53	2,5-piperazinedione, 3,6-bis(2-methylpropyl)	71.98	C_12_H_22_N_2_O_2_	226.17
15	32.788	0.67	2,5-piperazinedione, 3-benzyl-6-isopropyl-	83.72	C_14_H_18_N_2_O_2_	246.14
16	34.955	13.18	Pyrrolo[1,2-a]pyrazine-1,4-dione, hexahydro-3-(phenylmethyl)-	86.66	C_14_H_16_N_2_O_2_	244.12
17	38.174	2.76	Pyrimidine-2(1H)-thione, 4,4,6-trimethyl-1-(1-phenylethyl)-	20.61	C_15_H_19_N_2_S	259.13
18	38.995	6.75	Cyclo-(l-leucyl-l-phenylalanyl)	11.98	C_15_H_20_N_2_O_2_	260.15
19	39.917	1.97	p-(methylthio)benzyl alcohol	19.23	C_8_H_10_SO	154.05

## Discussion

The genus *Halomonas* was first described by Vreeland et al. in 1980 [23]. It belongs to the phylum *Proteobacteria*, class *Gammaproteobacteria*, order *Oceanospirillales*, and family *Halomonadaceae*. Among the species represented in the genus *Halomonas* are *H. denitrificans*, *Halomonas salaria* sp. nov, *Halomonas gomseomensis* sp. nov, and *Halomonas janggokensis* sp. nov [24]. *Halomonas*, also known as *H. huangheensis* [25], *Halomonas nitroreducens*, according to González-Domenech et al. (2008) [26]; *Halomonas litopenaei* was identified by Xue et al. (2018) [27], while *Halomonas urmiana* was identified by Khan et al. (2020) [28]. Most of them are halophilic and halotolerant bacteria that can survive at concentrations of 7-12% NaCl. *Halomonas* sp. are aerobic, gram-negative, non-spore-forming rods that measure 0.6-0.8 × 1.2-1.6 μm. Their colonies are smooth, translucent, round with complete edges, and brownish-yellow in color. These cells test positive for the enzymes catalase and oxidase, and they are motile because they have peritrichous flagella. Their growth is noted at pH levels between 7 and 10, with pH 8 to 9 being the ideal range, and temperatures between 5 and 50°C (with an optimum at 25 to 35°C). Furthermore, salinities between 2% and 20% NaCl are favorable for *Halomonas* sp. growth (the ideal range is between 8% and 10% NaCl). The Voges-Proskauer test produces negative results, and they neither produce indole nor hydrogen sulfide (H2S). Additionally, they can lower nitrite and nitrate [24].

In a similar vein, the powerful bacteria form round, translucent, flat colonies that are pale yellow in color, and gram-negative rods are arranged in light microscopy following gram staining. The biochemical tests show that urease is positive, hydrogen sulfide production is negative, oxidase and catalase are positive, and indole is negative. The powerful halophilic bacterium was identified as *Halomonas* sp. After molecular identification, the bacterium was found to have high similarities with *H. denitrificans*, *H. shengliensis*, *H. huangheensis*, *H. binhaiensis*, *H. cupid*, *H. stenophila*, and *H. pacifica*. Poly(3-hydroxybutyrate) (PHB), a metabolite linked to PHB accumulation during various log phases of bacterial growth, is produced by the *Halomonas* sp. These results imply that the utilization of *Halomonas* sp. KM-1 in the production of PHB exhibits multicomponent and phase-specific mechanisms [29]. El-Garawani et al. (2020) [6] reported that a recently isolated strain of *Halomonas* sp. (HA1) exhibits anticancer potential by inducing apoptosis and G2/M arrest in hepatocellular carcinoma (HepG2) cell line. According to a study by Youssif et al. (2020) [30], extracellular polymeric substances (EPS) were produced by the marine microorganism namely *Halomonas* sp. non-alcoholic steatohepatitis (NASH) in a medium containing 4M NaCl, pH 9, and a 7% initial inoculum. Additionally, the EPS demonstrated antimicrobial activity against a range of pathogens, with the greatest antibacterial activity demonstrated against *P. aeruginosa* and *S. aureus*, as well as *Bacillus subtilis*, *E. coli*, *P. aeruginosa*, and *C. albicans*. According to Youssif et al. (2020) [30], the EPS demonstrated antioxidant and anti-inflammatory activity, indicating the significance of using it for the treatment of chronic diseases where oxidative stress and inflammation play important roles. El-Garawani et al.'s recent study from 2020 [6] demonstrates that the marine isolate of *Halomonas* sp. exhibited anticancer activity against HepG2 liver cancer cells. *Halomonas* sp. produces a variety of metabolites, such as di-peptides and biosurfactants, which have anticancer properties. In particular, El-Garawani et al. (2020) [6] identified two biosurfactants as the most potent compounds: Surfactin C14 and Surfactin C15. The secondary metabolites produced by *S. aureus*, *P. aeruginosa*, and *C. albicans* exhibit moderate inhibition in the current study. These metabolites also exhibit antioxidant properties at higher concentrations and have high biological compatibility with human blood samples. However, more research is needed to fully understand the pharmacological activity and to optimize the culture media for the production of secondary metabolites, as well as for their purification and characterization.

Limitations of the study

While this study shows promising results, it's important to acknowledge some limitations. The observed antimicrobial activity of the secondary metabolites was moderate, and further research is needed to enhance their potency. Additionally, the study focused on a limited number of clinical pathogens, so evaluating their efficacy against a wider range of microorganisms is crucial. Analyzing the crude extract limits the understanding of individual compound activities, making it essential to isolate and characterize these compounds. Furthermore, the study's in vitro nature necessitates in vivo studies to assess the metabolites' true efficacy and safety. Finally, the potential long-term effects, such as resistance development or toxicity, remain unexplored and require further investigation. Addressing these limitations will provide a more comprehensive understanding of the therapeutic potential of these secondary metabolites.

## Conclusions

*Halomonas* sp. was found to be the potent bacterium isolated from Saltpan, Tamil Nadu, India. It exhibits moderate inhibition against *S. aureus* and *E. coli* for both the well diffusion and MIC assays, as well as moderate antioxidant properties for both intracellular and extracellular metabolites. Blood samples and the metabolites generated by *Halomonas* sp. are highly compatible. It yields 23 distinct compounds, of which seven are major; however, based on 96% similarity, one molecule may be identical to its name. Therefore, additional mass production is needed to purify and analyze the molecule responsible for this microbial and antioxidant activity and to validate its efficacy against various pathogens and its mode of action, based on the microbial activity of secondary metabolites and compounds produced by this bacterium.
